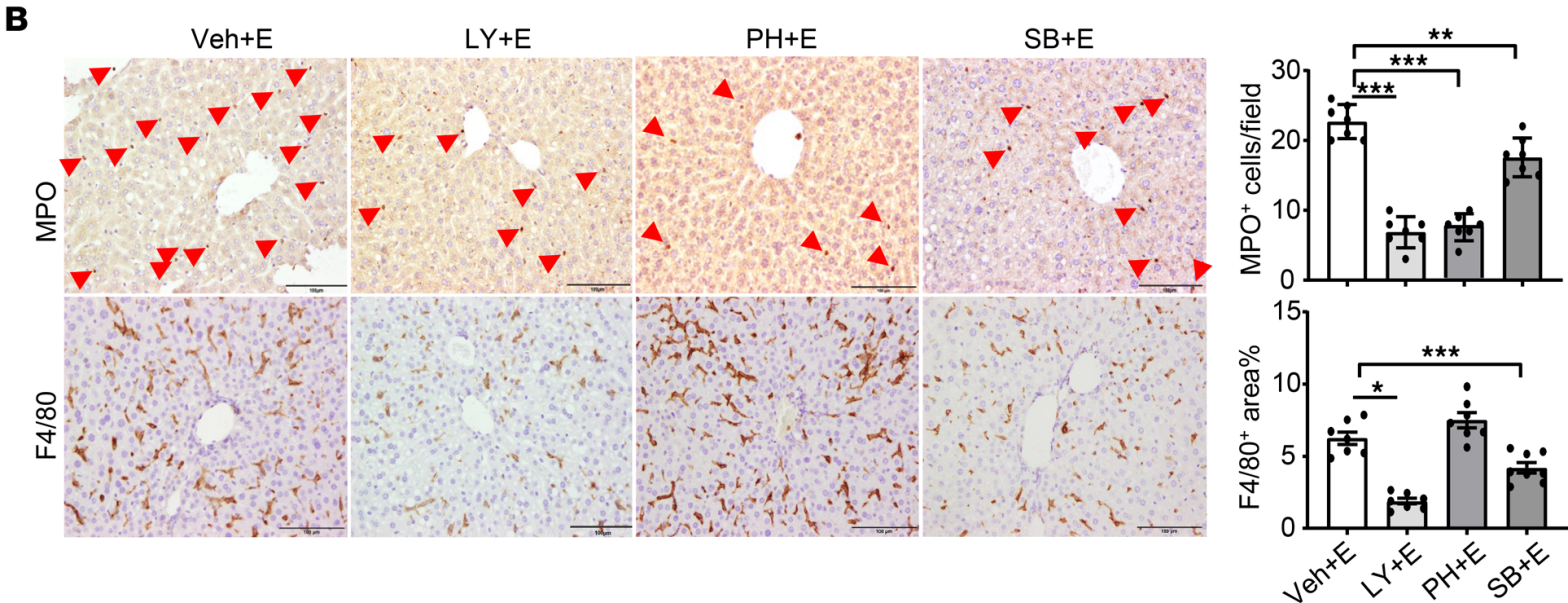# Chronic-plus-binge alcohol intake induces production of proinflammatory mtDNA-enriched extracellular vesicles and steatohepatitis via ASK1/p38MAPKα﻿﻿-dependent mechanisms

**DOI:** 10.1172/jci.insight.197929

**Published:** 2025-08-22

**Authors:** Jing Ma, Haixia Cao, Robim M. Rodrigues, Mingjiang Xu, Tianyi Ren, Yong He, Seonghwan Hwang, Dechun Feng, Ruixue Ren, Peixin Yang, Suthat Liangpunsakul, Jian Sun, Bin Gao

Original citation: *JCI Insight*. 2020;5(14):e136496. https://doi.org/10.1172/jci.insight.136496

Citation for this corrigendum: *JCI Insight*. 2025;10(16):e197929. https://doi.org/10.1172/jci.insight.197929

The authors recently became aware that the GS+E panel in Figure 4B is the same as the LY+E panel in Figure 7B. The error was inadvertent and occurred during the revision process. The correct figure panels are shown below. The HTML and PDF files have been updated.

The authors regret the error.

## Figures and Tables

**Figure 4B:**
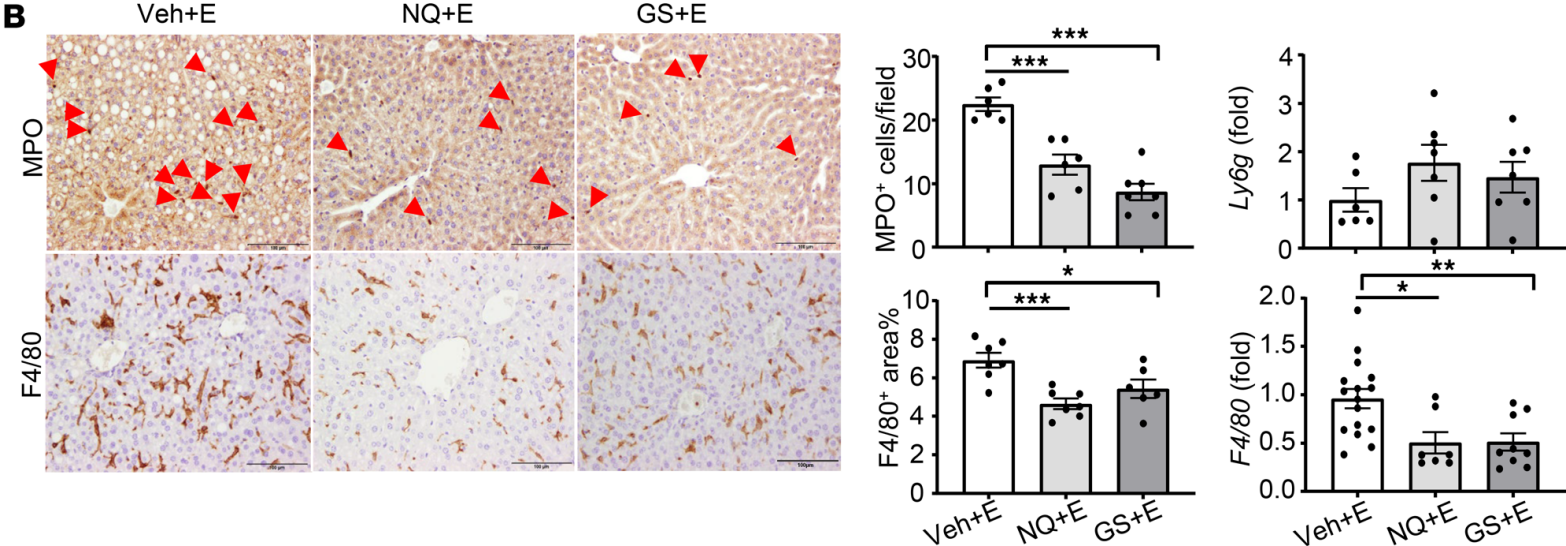


**Figure 7B:**